# Development of mediastinal lymphoma after radiotherapy for concurrent medulloblastoma and PNET in a patient with Gorlin syndrome

**DOI:** 10.1186/s12957-016-0967-5

**Published:** 2016-08-12

**Authors:** Tao Jiang, Junmei Wang, Ying Wang, Chunde Li

**Affiliations:** 1 Department of Neurosurgery, Beijing Tiantan Hospital, Capital Medical University, 6 Tiantan xili, Chonwen District, Beijing, 100050 China; 2 Beijing Neurosurgical Institute, Capital Medical University, Beijing, 100050 China; 3 Beijing Chao-Yang Hospital, Capital Medical University, Beijing, 100020 China

**Keywords:** Medulloblastoma, Gorlin syndrome, Chemotherapy, Café-au-lait spots, SHH subtype

## Abstract

**Background:**

Very young children with Gorlin syndrome are at risk for developing medulloblastoma. Patients with Gorlin syndrome may have multiple system abnormalities, including basal cell carcinomas, jaw cysts, desmoplastic medulloblastoma, palmar/plantar pits, rib abnormalities, and intracranial falx calcification. The early diagnosis of Gorlin syndrome in desmoplastic medulloblastoma patients is very important because these patients should receive chemotherapy as a first-line treatment and should avoid radiotherapy as much as possible.

**Case presentation:**

In the present study, a 5-year-old male patient had a concurrent cerebellar desmoplastic medulloblastoma and temporal primitive neuroectodermal tumor. Examinations of this patient revealed multiple café-au-lait spots, a jaw cyst, and a bifid rib. A molecular classification analysis revealed that the patient’s cerebellar tumor was of the sonic hedgehog subtype. Twenty-seven months after tumor resection and cerebrospinal irradiation were performed, mediastinal lymphoma was found in the patient. The patient ultimately died of lymphoma. To the best of our knowledge, this is the first report of a concurrent medulloblastoma and primitive neuroectodermal tumor and the fourth report of multiple café-au-lait spots in a patient with Gorlin syndrome. This report is also the first account of the development of mediastinal lymphoma after spinal irradiation in a patient with Gorlin syndrome.

**Conclusions:**

Chemotherapy should be the first-line treatment for medulloblastoma patients with Gorlin syndrome. Young patients with medulloblastoma of the desmoplastic subtype and multiple café-au-lait spots should be thoroughly examined for Gorlin syndrome.

## Background

Gorlin syndrome (GS), also known as basal cell nevus syndrome (BCNS, OMIM #109400), basal cell carcinoma nevus syndrome (BCCNS), and Gorlin-Goltz syndrome, is an autosomal inherited syndrome that was first described in 1963 [1]. The prevalence rates of GS range from 1/55,600 to 1/30,827 in the UK, 1/164,000 in Australia, and 1/235,800 in Japan [2]. GS is an autosomal genetic disorder that is generally caused by a mutation in the patched-1 homolog (PTCH1) gene, which has complete penetrance and a variable phenotype. This syndrome is characterized by the existence of multiple basal cell carcinomas (BCCs), jaw cysts, desmoplastic medulloblastoma, palmar/plantar pits, rib abnormalities, and intracranial falx calcification.

The presence of desmoplastic medulloblastoma (DMB) and a primitive neuroectodermal tumor (PNET) is currently the major criterion for the diagnosis of GS [3]. However, the early diagnosis of GS in DMB patients is difficult because the other criteria used to establish a diagnosis of GS, such as intracranial calcification and BCC, may not occur before the patient is 10 years old. Most medulloblastoma patients with GS are less than three years old, with a mean age of 2 years. Suspected DMB patients should be screened for GS because irradiation of GS patients may cause the development of radiation-induced tumors, such as meningioma and ependymoma. Chemotherapy is the first-line treatment for these patients. Here, we report a 5-year-old boy who was diagnosed with GS and a concurrent cerebellar medulloblastoma and temporal PNET, as well as multiple café-au-lait spots. Twenty-seven months after tumor resection and radiotherapy were performed, mediastinal lymphoma was found. To the best of our knowledge, this is the first report of this phenomenon.

## Case presentation

### History and examination

**Fig. 1:**
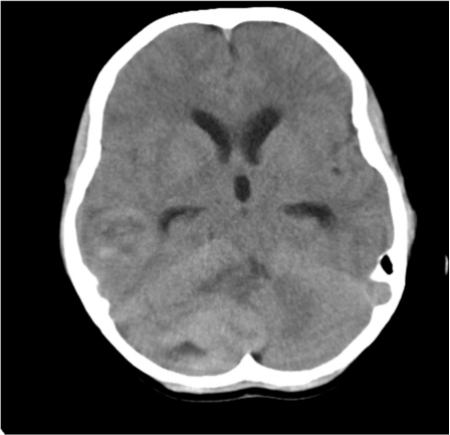
CT image of the 5-year-old boy. Two intracranial tumors were observed, a right temporal tumor and a right cerebellar tumor. The tumors had a round shape and were of high density

**Fig. 2:**
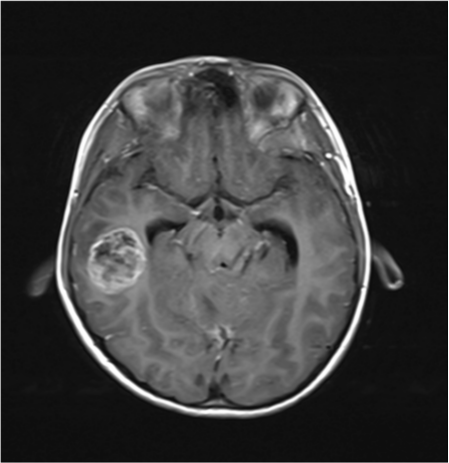
T1-weighted MRI image of the temporal tumor, with obvious contrast

**Fig. 3:**
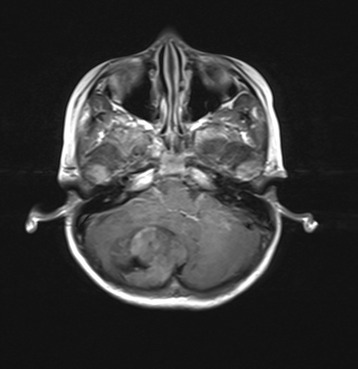
Image of the right cerebellar tumor, with moderate enhancement

A 5-year-old boy presented with headache, vomiting, and vertigo with a duration of 5 months. CT and MRI examinations revealed the presence of a right cerebellar mass with mild enhancement and of a right temporal mass with moderate enhancement (Figs. 1, 2, and 3). The tumors were hypointensive in T1-weighted MRI scans and hyperintensive in T2-weighted MRI scans. CT examination revealed that both tumors were hyperdense. Following these examinations, the patient was referred to our hospital.

### Surgery and diagnosis

**Fig. 4:**
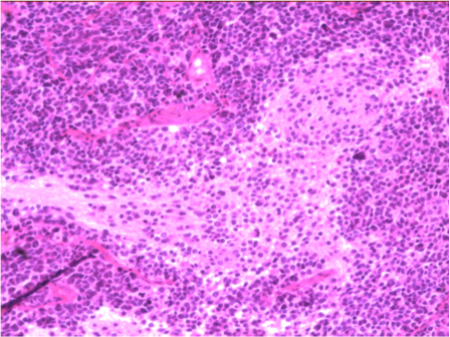
Microscope image of the right cerebellar tumor, which was of the desmoplastic subtype

**Fig. 5:**
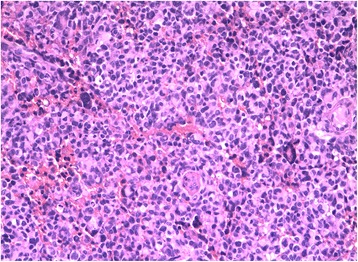
Microscope image of the right temporal tumor, which was a primitive ectodermal tumor

During surgery, the two tumors were observed to have similar appearances. Both tumors were reddish-colored and soft, had a moderate blood supply, and were easy to suction. The postsurgical pathology reports stated that the tumors were a DMB (cerebellar mass) and PNET (temporal mass) (Figs. 4 and 5).

**Fig. 6:**
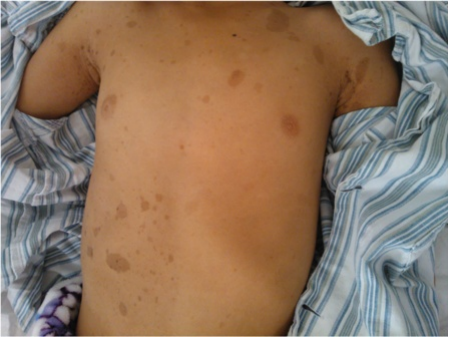
Image showing multiple café-au-lait spots distributed over the body

**Fig. 7:**
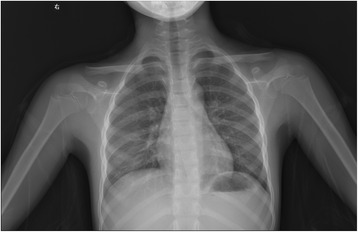
Image showing that the right fourth rib was a bifid rib

**Fig. 8:**
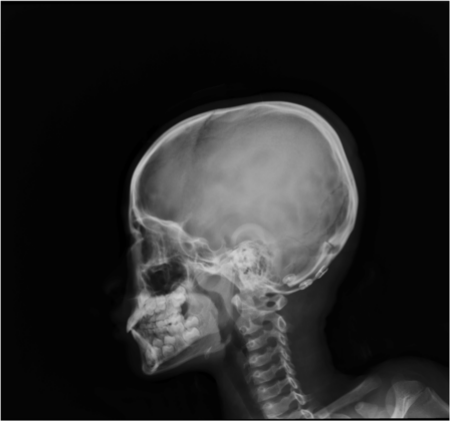
Image of the jaw cyst

Due to the suspicion that the boy had GS, he was evaluated for this condition. The circumference of his head was 48 cm. Physical examination revealed the presence of multiple café-au-lait spots (Fig. 6). Plain film X-ray imaging demonstrated the presence of a bifid rib and a jaw cyst (Figs. 7 and 8). The PTCH1 gene test was negative. We conducted a molecular classification of the cerebellar tumor using the real-time polymerase chain reaction (PCR) method [4] and the NanoString method [5] and discovered that the DMB was a SHH subtype tumor. Based on two major and one minor criteria for GS (desmoplastic MB, bifid rib, and jaw cyst, respectively), an unambiguous diagnosis of GS was made.

### Radiotherapy and post-treatment course

**Fig. 9:**
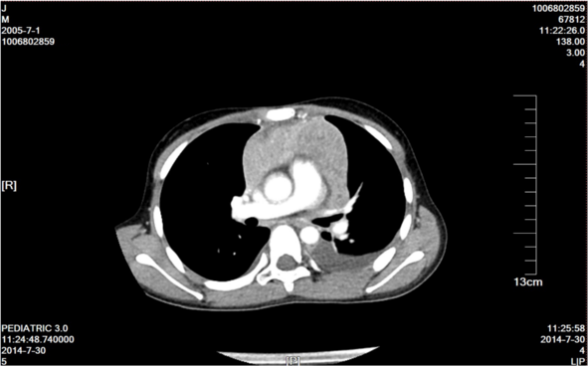
Image showing the mediastinal mass with thoracic effusion

**Fig. 10:**
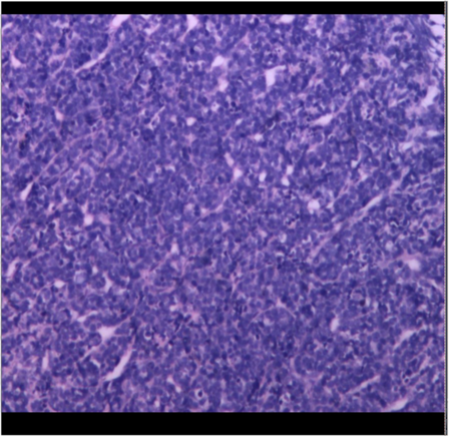
Microscope image of the thoracic tumor, which was a T cell non-Hodgkin’s lymphoma

After the patient was discharged, his parents refused to allow him to receive chemotherapy, which is a treatment that must be covered by out-of-pocket payments in China, due to their financial difficulties. The patient instead underwent 30.6-Gy irradiation of the entire brain and the spinal axis and 54-Gy irradiation of the posterior fossa. At follow-up, an MRI examination showed no tumor recurrence. Twenty-seven months after receiving radiotherapy, the patient experienced chest pain and had a fever. CT examinations revealed the presence of a mediastinal mass and chest effusion (Fig. 9). Analysis of a biopsy performed at another hospital demonstrated that the mass was T cell non-Hodgkin’s lymphoma (Fig. 10). The patient’s parent refused treatment because of financial difficulties, and the boy died 1 month later.

### Discussion

DMB and medulloblastoma with extensive nodularity (MBEN) are closely associated with GS. The development of DMB and MBEN, which are generally the first tumoral manifestations in patients with GS, is thought to be the major criterion for the diagnosis [3]. The prevalence of MB in GS patients in early childhood is difficult to estimate. The incidence of GS in MB patients was reported to be 1–2 %, and 3–5 % of GS patients develop medulloblastoma, generally within the first 2 years of life [6]. In a retrospective investigation, 5 of 82 medulloblastoma patients were diagnosed with GS [7]. In Amlashi’s cohort of 76 MB patients, the incidence of GS among the entire cohort was 4 %, the incidence of GS in patients younger than 5 years old was 10.7 %, and the incidence of GS in patients younger than 2 years old was 25 % [8]. In a Japanese survey, 3.3 % of 157 GS patients had MB [2].

To the best of our knowledge, this is the first report of a concurrent infratentorial medulloblastoma and a supratentorial PNET in a GS patient. On cerebrospinal axis MRI examination, there were no signs of CSF seeding; two images appeared different under microscope examination, which excluded the occurrence of tumor metastasis. The molecular classification of the DMB as a SHH subtype tumor was also consistent with the diagnosis of GS. Our patient could have been diagnosed with GS based on the presence of DMB, a PNET, a jaw cyst, a bifid rib, and multiple café-au-lait spots, as well as the classification of the DMB as a SHH subtype tumor. The occurrence of multiple café-au-lait spots is associated with many hereditary disease, including neurofibromatosis type 1, McCune-Albright syndrome, Cowden syndrome, and LEOPARD syndrome [9]. This is the fourth case report of multiple café-au-lait spots in a GS patient [9, 10]. Because the clinical diagnostic criteria for GS are continually changing, we propose that the presence of café-au-lait spots in young DMB patients should be considered a “trigger” for ordering a diagnostic evaluation and a molecular blood test for GS.

GS patients are at a high risk of developing multiple BCCs and other radiation-induced tumors, such as meningioma, ependymoma, and fibrosarcoma, in irradiated areas. To date, this is the first report of the development of post-treatment non-Hodgkin’s lymphoma in a GS patient. The hedgehog pathway regulates intrathymic T cell development. Aberrant activation of the hedgehog pathway is associated with the pathogenesis of malignant lymphoma [11]. Irradiation induces DNA damage and genomic instability in circulating and thymic lymphocytes, which results in apoptosis, abnormal DNA methylation, and changes in RNA expression [12, 13]. Our patient developed mediastinal lymphoma, which was unequivocally diagnosed as a radiation-induced tumor. Interestingly, we found one report of a radiation-induced PNET that developed following treatment for non-Hodgkin’s lymphoma [14]. These findings may facilitate elucidation of the molecular mechanisms underlying tumorigenesis in GS patients.

Early and prompt diagnosis is important in patients suspected to have GS, as chemotherapy is the first-line treatment for tumors in GS patients. The desmoplastic variant of MB and MBEN in GS generally occur in children who are 2 years of age or younger. Most of the main criteria for GS, such as intracranial calcification, jaw cysts, and BCC, do not appear until the second decade of life, which makes early diagnosis of GS in very young patients difficult [15]. Medulloblastoma patients with GS generally have a promising survival rate due to recent advancements made in chemotherapy [16]. The detection rate of a mutated PTCH 1 gene is only 50–85 % [17], which makes early diagnosis more difficult. Amlashi et al. have even suggested avoiding radiotherapy in DMB patients who are less than 5 years old [8].

The overexpression of the members of the canonical hedgehog signaling pathway plays an important role in tumorigenesis in GS patients. In the majority of GS patients, the loss of function of PTCH1 has been found, which causes the reduction of the inhibition of the smoothened (SMO) oncogene and the subsequent aberrant activation of the glioma-associated oncogene homolog (GLI) family members. It is possible that SMO inhibitors, such as vismodegib, may serve as new therapeutics for the treatment of tumors in GS patients. Vismodegib has proven to be effective in the treatment of GS-related BCC and keratocystic odontogenic tumors [18, 19]. Robinson et al. reported that vismodegib exhibited activity against adult recurrent or refractory SHH-MB [20]. However, the response to SMO inhibitors of medulloblastoma patients was variable and transient, and this drug was most effective in treating tumors with upstream activating aberrations in the SHH pathway. The existence of a PTCH1 mutation was correlated with a positive response to the drug, and aberrations in GIL2 and SUFU were found in the nonresponders [20].

The lack of efficacy of SMO inhibitors and the acquired resistance to these inhibitors in medulloblastoma patients argues for the use of GLI-specific inhibitors. GLI1 is the most significant member of the hedgehog pathway and plays a role in promoting carcinogenesis. Several studies have shown that aberrant GLI1 expression occurred independently from the signaling of the canonical HH pathway through PTCH and SMO [21, 22] and was responsible for the development of radioresistance and chemoresistance in tumors [23]. The aberrant expression of GLI1 was closely linked to the activity of several non-canonical signaling pathways, such as the Kirsten rat sarcoma viral oncogene homolog (KRAS) pathway, the avian myelocytomatosis virus oncogene cellular homolog (C-MYC) pathway, the transforming growth factor β (TGFβ) pathway, the wingless-type MMTV integration site family (WNT) pathway, and the β-catenin pathway. Together, these data suggest that administering specific inhibitors of the final step in the hedgehog pathway may be the most effective treatment option and the ideal approach to use in future studies. Currently, there are several agents (HPT, GANT58, GANT61, and arsenic trioxide) that are known to inhibit the transcriptional activity of GLI [21, 24]. Although GLI1-specific inhibitors are still in the preclinical stage of testing, studies in which combinations of GLI1 inhibitors and chemotherapeutic agents were used to treat other types of tumors have been conducted [24].

## Conclusions

To the best of our knowledge, this is the first report of a concurrent medulloblastoma and PNET and the fourth report of multiple café-au-lait spots in a patient with GS. This is also the first report of mediastinal lymphoma developing after spinal irradiation for the treatment of GS. Chemotherapy should be the first-line treatment for medulloblastoma patients with GS. We propose that young patients with the desmoplastic subtype of medulloblastoma and multiple café-au-lait spots should be thoroughly examined for the existence of GS.

## Abbreviations

BCC: basal cell carcinoma; CSI: cerebrospinal axis irradiation; DMB: desmoplastic medulloblastoma; GLI: glioma-associated oncogene homolog; GS: Gorlin syndrome; MBEN: medulloblastoma with extensive nodularity; PNET: primitive neuroectodermal tumor; SMO: smoothened
